# Boosting Photocatalytic Performance of ZnO Nanowires via Building Heterojunction with Conjugated 2,4,6-Triaminopyrimidine-g-C_3_N_4_

**DOI:** 10.3390/molecules29163716

**Published:** 2024-08-06

**Authors:** Jiahui Lou, Lihong Wang, Yaqiong Huang, Jun Xing, Xiaojie Yang

**Affiliations:** 1 Hubei Key Laboratory of Radiation Chemistry and Functional Materials, School of Nuclear Technology and Chemistry & Biology, Hubei University of Science and Technology, Xianning 437100, China; 2 School of Biomedical Engineering and Imaging, Hubei University of Science and Technology, Xianning 437100, China

**Keywords:** 2,4,6-triaminopyrimidine, g-C_3_N_4_, conjugate, photocatalytic performance

## Abstract

Photocatalysis is one of the most effective ways to solve environmental problems by solving pollutants. This article designed and prepared a conjugated system of 2,4,6-triaminopyrimidine-g-C_3_N_4_ (TAP-CN) to modify ZnO NWs. We systematically studied the photocatalytic performance of ZnO NWs modified with different ratios of TAP-CN. The results showed that 9 wt% TAP-CN-30/ZnO NWs had the best degradation effect on Rhodamine B dye. The degradation rate was 99.36% in 80 min. The excellent degradation performance was attributed to the TAP-CN conjugated system promoting photo-generated charge transfer. This work provided guidance for designing efficient composite catalysts for application in other renewable energy fields.

## 1. Introduction

Water pollution caused by organic pollutants is becoming one of the most serious threats to public health and safety. In order to remove pollutants from water, researchers use various methods to treat them. Among numerous methods, photocatalytic technology utilizes solar energy to degrade pollutants in wastewater, which has attracted widespread attention from researchers [1,2,3,4,5]. ZnO has received widespread attention and research due to its low cost and high stability [6,7,8]. Zinc oxide absorbs visible or ultraviolet light, forms excited states on the surface, and forms electron hole pairs. The separation of electron hole pairs and the combination of active oxides promote the degradation of organic pollutants. However, application of ZnO was limited due to its broad bandgap width [9,10]. Therefore, developing a photocatalyst that could decompose organic pollutants under visible light has become a research focus [11,12]. ZnO-based semiconductor composite materials have become one of the most effective methods to enhance the photocatalytic activity of ZnO [13,14,15].

The bandgap width of g-C_3_N_4_ was 2.4–2.8 eV has attracted extensive research due to its low cost, non-toxic, high stability, and optoelectronic performance [16,17,18]. g-C_3_N_4_ had drawbacks such as a fast recombination rate of photo-generated electron hole pairs and small surface area, which limited its application in photocatalysis [19,20,21,22]. Researchers mainly improved their photocatalytic activity through morphology and structural modifications [23,24,25,26,27]. Utilizing element doping, constructing conjugated systems, and donor-acceptor (D-A) systems, etc., enhanced the extraction and transfer of photo-generated electrons, increasing reaction active sites, etc. [28,29,30,31]. Abubshait et al. [32] prepared Co-dosed ZnO NPs (CoZnO)/polyvinyl alcohol hydrogel (PVA) composite materials to degrade organic pollutants (methyl orange, MO). The effect of Co doping on photocatalytic performance was systematically studied, and 12% Co-doped ZnO NPs (12CoZnO/PVA) showed the best degradation performance. The photocatalytic performance of the prepared 12CoZnO/PVA was significantly improved, and MO was completely degraded within 48 min. Huong et al. [33] reported a preparation method for carbon-doped ZnO, using an aqueous solution of mangosteen peel extract as a carbon source to prepare ZnO-C. The crystal structure of ZnO-C was optimized through calcination technology. The average size of ZnO-C nanoparticles prepared was 25–70 nm. The aqueous solution of bamboo peel extract served as a carbon source while achieving heteroatoms such as N, S, and P. The ZnO-C sample calcined at 700 °C for 1 h exhibited the best photocatalytic activity results. The prepared ZnO-C-700-1 almost completely degraded methylene blue and malachite green under photocatalytic action. In an alkaline environment, the degradation rate of Rhodamine B by the prepared material reached 62.13% within 120 min. Vijayan et al. [34] prepared efficient g-C_3_N_4_-ZnO nanocomposites with different weight percentages (25:75, 50:50, and 75:25) using a simple wet impregnation method. The photocatalytic performance of 75 wt% g-C_3_N_4_-25 wt% ZnO nanocomposites exhibit higher photocatalytic activity. The excellent photocatalytic performance is mainly attributed to the improvement of light absorption in the visible light region. The prepared composite material effectively reduces the recombination of charge carriers. Guo et al. [35] prepared Z-type Ti, Ga co-doped ZnO/g-C_3_N_4_ (TGZ/CN) (Ti, Ga co-doped ZnO, TGZ) heterostructure photocatalysts by using the simple sol-gel method and single-phase dispersion method. The TGZ/CN composite material exhibited higher photocatalytic performance for the degradation of MB, with an effective photocatalytic degradation rate of 95.4% within 105 min under visible light. The improvement in photocatalytic performance was mainly attributed to the effective reduction of the bandgap of ZnO after Ti/Ga co-doping, as well as the promotion of separation by the construction of Z-type heterojunctions and the photoexcitation of e^−^/h^+^ pairs.

This article constructed a π conjugated system of TAP-CN through the thermal polymerization method and combined it with ZnO nanowires prepared by the thermal decomposition method to form a TAP-CN/ZnO NWs heterostructure. The introduction of conjugated systems was conducive to the separation and transfer of photo-generated electrons and hole pairs. The as-prepared TAP-CN formed a heterojunction at the interface with ZnO NWs, which facilitated carrier transport and improved photocatalytic performance. The results showed that when TAP doping was 30 mg and TAP-CN-30 doping ratio was 9 wt%, 9 wt% TAP-CN-30/ZnO NWs exhibited the best catalytic performance. The degradation rate of Rhodamine B (RhB) solution (20 mg L^−1^) reached 99.36% in 80 min.

## 2. Experiment

### 2.1. Preparation of ZnO Nanowires (ZnO NWs)

ZnO NWs were prepared by the solid-state thermal decomposition method. First, 14 g Zn(CH_3_COO)_2_·2H_2_O was ground in an agate mortar for 10 min and placed in a corundum crucible. The crucible was transferred to a muffle furnace and heated at a heating rate of 2 °C·min^−1^ to 300 °C, followed by a constant temperature of 120 min. After cooling to room temperature, ZnO NWs was obtained.

### 2.2. Preparation of g-C_3_N_4_

Exactly 70 g of urea (CH_4_N_2_O) was placed in an alumina crucible and heated in a muffle furnace at a heating rate of 5 °C min^−1^ to 550 °C before calcination for 180 min. After calcination and grinding, light yellow g-C_3_N_4_ powder was obtained.

### 2.3. Preparation of 2,4,6-Triaminopyrimidine-g-C_3_N_4_ (TAP-CN) and TAP-CN/ZnO NWs

**Figure 1:**
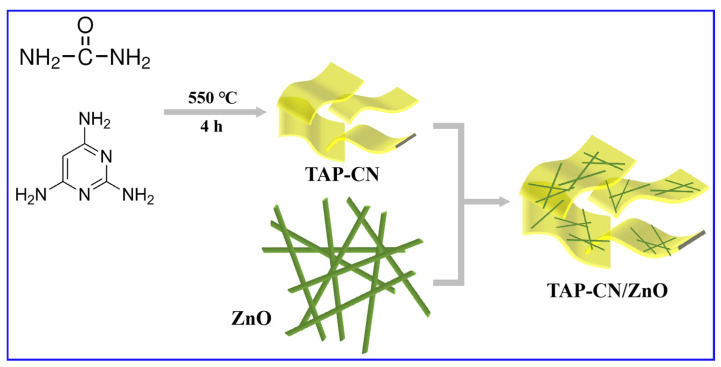
Schematic diagram of the experimental preparation process.

Together, 10 g of urea and 2,4,6-triaminopyrimidine (x = 10 mg, 30 mg, 50 mg) were ground and mixed in a corundum crucible at 550 °C (at rate of 17 °C·min^−1^) for 4 h. The product was named TAP-CNx (x = 10 mg, 30 mg, 50 mg). Then, 0.1 g ZnO NWs was added with the required mass of TAP-CN-30 (with doping amounts of 3 wt%, 6 wt%, 9 wt%, and 12 wt%, respectively). We then added anhydrous ethanol, stirred for 8 h, and transferred the mixture to a 60 °C oven for 48 h. The TAP-CN/ZnO composite materials obtained are in Figure 1.

## 3. Results and Discussion

### 3.1. Morphological and Structural Characterization

**Figure 2:**
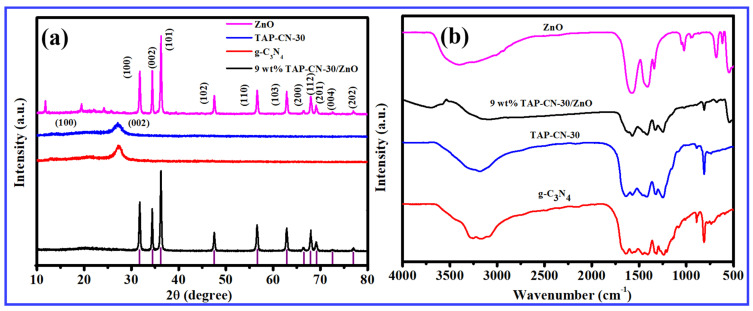
(**a**) XRD patterns of ZnO, TAP-CN-30, g-C_3_N_4_, 9 wt% TAP-CN-30/ZnO; (**b**) infrared characterization of ZnO, TAP-CN-30, g-C_3_N_4_, 9 wt% TAP-CN-30/ZnO.

Figure 2a was the XRD spectrum of g-C_3_N_4_ and ZnO NWs, TAP-CN-30, and 9 wt% TAP-CN-30/ZnO. The XRD patterns of g-C_3_N_4_ and TAP-CN-30 had two diffraction peaks located around 13.0° and 27.3°, corresponding to the (100) and (002), respectively. These peaks were attributed to the layered and interlayer stacking structures of the aromatic system of carbon nitride, respectively, which was the characteristic peak of g-C_3_N_4_. The XRD pattern of 9 wt% TAP-CN-30/ZnO not only had characteristic peaks in g-C_3_N_4_, but also was accompanied by characteristic peaks in ZnO, located at 31.78°, 34.47°, 36.28°, 47.55°, 56.66°, 62.98°, 66.49°, 68.03°, 69.20°, 72.62°, and 76.95° correspond to (100), (002), (101), (102), (110), (103), (200), (112), (201), (004), and (202), respectively (JCPDS NO.75-0576). XRD showed the presence of characteristic peaks of g-C_3_N_4_ and ZnO in the 9 wt% TAP-CN-30/ZnO composite material, indicating the successful preparation of the composite material. Figure 2b showed the FT-IR spectra of g-C_3_N_4_, ZnO NWS, TAP-CN-30, and 9 wt% TAP-CN-30/ZnO. The peaks of g-C_3_N_4_ at 1242.48 cm^−1^ and 1640.73 cm^−1^ corresponded to C-N and C=N stretching vibrations in aromatic carbon nitrogen heterocycles, respectively. The peak at 814.52 cm^−1^ was attributed to the stretching vibration peak of the s-triazine ring structure, which was consistent with the characterization results of later XRD, further confirming the graphene-like structure of g-C_3_N_4_. The multiple peaks of TAP-CN-30 in the range of 1200 cm^−1^ to 1600 cm^−1^ corresponded to the stretching vibrations of typical aromatic C-N heterocycles, while the wide peaks in the range of 3000–3500 cm^−1^ were attributed to the terminal amino groups and surface adsorbed H_2_O. The characteristic peak positions of the carbon–nitrogen heterocycles added to TAP remain unchanged. The results indicated that the incorporation of TAP did not significantly alter the core chemical framework of carbon nitride. 9 wt% TAP-CN/ZnO exhibited a weak absorption peak at 548.27 cm^−1^, due to ZnO stretching vibration, indicating the formation of ZnO crystals. The FT-IR spectra of 9 wt% TAP-CN-30/ZnO was similar to the characteristic spectra of g-C_3_N_4_ and ZnO, demonstrating the successful recombination of TAP-CN-30 with ZnO NWs while retaining typical graphite structures.

**Figure 3:**
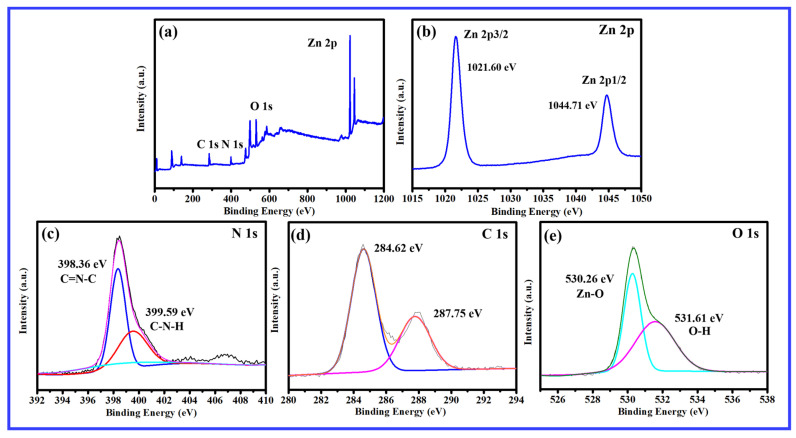
XPS spectra of 9 wt% TAP-CN-30/ZnO: (**a**) survey; (**b**) Zn 2p; (**c**) N 1s; (**d**) C 1s; (**e**) O 1s.

As shown in Figure 3, the composite material contains four elements: Zn, O, C, and N, and there were no impurity peaks in Figure 3a, indicating the successful synthesis of g-C_3_N_4_/ZnO NWs composite material. In Figure 3b, Zn^2+^ was located at two peaks of 1045.2 eV (Zn 2p_1/2_) and 1022.0 eV (Zn 2p_3/2_). In Figure 3c, a strong characteristic peak of N 1s appears at 399.1 eV and 400.1 eV, corresponding to C-N=C and N-(C)3. The binding energies of 284.7 eV and 288.5 eV corresponding to the C-C and C=N bonds were observed in the C 1s peak spectrum, as shown in Figure 3d. As shown in Figure 3e, The characteristic peak position of O 1s was 531.1 eV corresponds to O^2−^ in ZnO. XPS characterization further indicated the successful recombination of ZnO NWs and TAP-CN.

**Figure 4:**
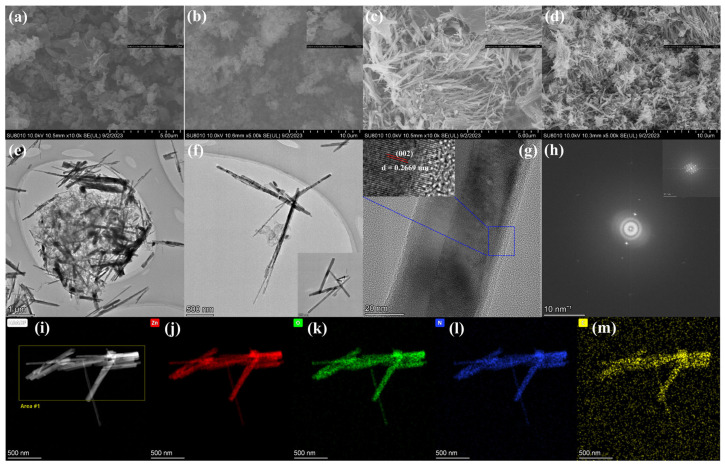
FESEM images: (**a**) SEM images of g-C_3_N_4_; (**b**) SEM images of TAP-CN-30; (**c**) SEM images of ZnO NWs; (**d**) SEM image of 9 wt% TAP-CN-30/ZnO; TEM images: (**e**,**f**) 9 wt% TAP-CN-30/ZnO; HRTEM images: (**g**,**h**) 9 wt% TAP-CN-30/ZnO; (**i**–**m**) EDS mapping images of 9 wt% TAP-CN-30/ZnO.

Morphological analysis was conducted on g-C_3_N_4_, TAP-CN-30, ZnO NWs, and 9 wt% TAP-CN-30/ZnO samples using SEM. TAP-CN-30 was prepared by thermal condensation polymerization, as shown in Figure 4b. The as-prepared TAP-CN-30 exhibited obvious small particle aggregation morphology, and its surface particles was more pronounced compared to the block like structure of g-C_3_N_4_. Compared with the SEM images of g-C_3_N_4_ and TAP-CN-30, it can be seen that TAP-CN-30 had a thinner nanosheet structure. The introduction of TAP did not damage the morphology of carbon nitride nanosheets, and the structure of carbon nitride nanosheets was still maintained in TAP-CN-30. The ZnO prepared by the solid-state thermal decomposition method presents nanowires, as shown in Figure 4c. The as-prepared 9 wt% TAP-CN-30/ZnO showed that ZnO NWs was uniformly distributed in TAP-CN-30, which was conducive to the formation of heterojunctions to enhance photocatalytic performance in Figure 4d. In order to further investigate the microstructure of 9 wt% TAP-CN-30/ZnO, TEM characterization was performed in Figure 4e–h. The prepared 9 wt% TAP-CN-30/ZnO was performed by mapping analysis in Figure 4i–m. It can be seen that C and N elements were uniformly distributed on the surface of ZnO nanowires. It indicated that the prepared TAP-CN-30 was uniformly distributed on the surface of ZnO nanowires. Combined with XPS, it can be seen that TAP-CN-30 was chemically bonded to ZnO, forming a heterojunction on the surface. It was consistent with XRD and TEM characterization.

### 3.2. Evaluation of Photocatalytic Degradation Performance

RhB was used as a simulated pollutant to explore the photocatalytic performance of the catalyst under visible light irradiation. Firstly, 50 mg of zinc oxide and 50 mL of RhB (20 mg/L) solution were placed in a test tube. Then, the test tubes were placed into the photocatalytic reaction device and stirred in the dark for 30 min to reach the adsorption-desorption equilibrium between the photocatalyst and RhB solution. Afterwards, the xenon lamp was turned on and the photocatalytic degradation experiment was carried out under visible light.

The degradation of RhB was monitored by observing the decrease in the intensity of the characteristic absorption peak at 546 nm with time. The degradation reaction kinetics of RhB can be expressed quantitatively with the use of the pseudo-first-order model: −ln(C_t_/C_0_) = f(t), where C_0_ referred to the original dye concentration; C_t_ referred to the RhB concentration; K_0_ represented the pseudo-first-order rate constant; and t represented the irradiation time. In accordance with this equation, K_0_ can be acquired from a linear plot of ln(C_0_/C_t_) against t, which represents the degradation rate and is proportional to the photocatalytic degradation rate.

**Figure 5:**
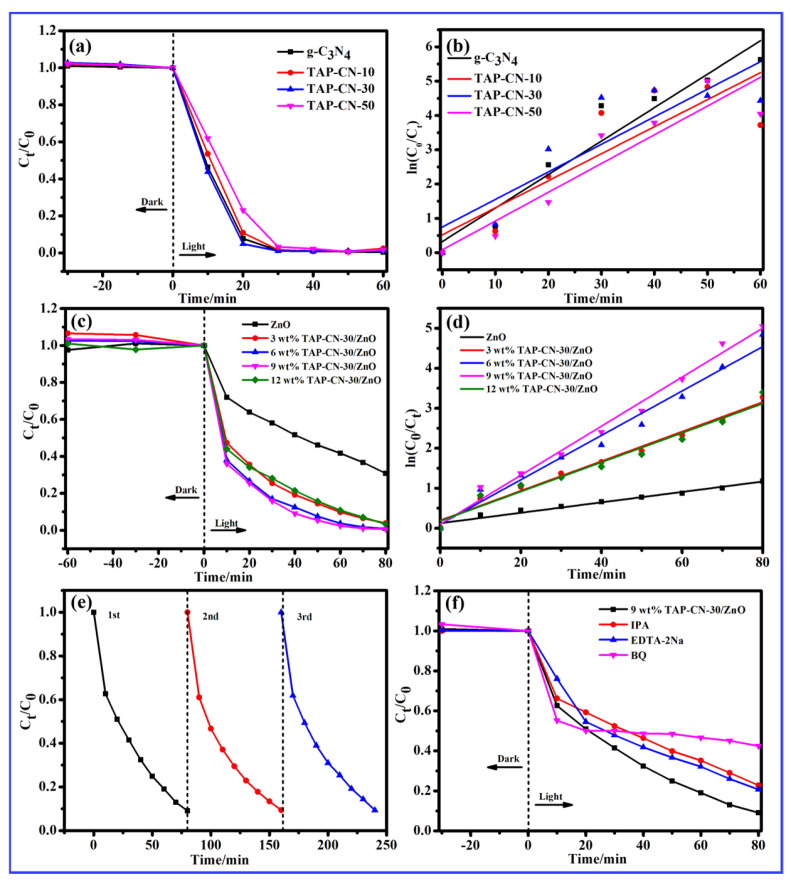
(**a**) Photocatalytic activity of TAP-CN for photocatalytic degradation of RhB in visible light; (**b**) Reaction kinetic curve of RhB photocatalytic degradation by TAP-CN under visible light irradiation; (**c**) Photocatalytic activity of TAP-CN-30/ZnO for photocatalytic degradation of RhB in visible light; (**d**) Reaction kinetic curve of RhB photocatalytic degradation by TAP-CN-30/ZnO under visible light irradiation; (**e**) 9 wt% TAP-CN-30/ZnO degradation RhB cycle test diagram; (**f**) Capture experimental diagram of RhB related active substances degraded by 9 wt% TAP-CN-30/ZnO.

The photocatalytic activity of pure g-C_3_N_4_ and TAP-CNx (x = 10 mg, 30 mg, 50 mg), as well as TAP-CN/ZnO (3 wt%, 6 wt%, 9 wt%, 12 wt%) composite materials were investigated for the photodegradation of RhB solution under visible light irradiation at room temperature. Figure 5a,c investigated the effects of TAP doping in TAP-CN composite materials and TAP-CN-30 doping in TAP-CN/ZnO on the photocatalytic degradation of RhB. The results showed that when TAP-CNx (x = 10 mg, 30 mg, 50 mg) x = 30 mg, the degradation efficiency was 98.81% within 60 min, and the sample had the best photocatalytic degradation ability. The photocatalytic degradation effect of TAP-CNx (x = 10 mg, 30 mg, 50 mg) composite material on RhB was significantly better than that of g-C_3_N_4_. The optimal doping amount of TAP was 30 mg. The introduction of TAP increased the conductivity of the material, which was conducive to the rapid arrival of photo-generated charge carriers at the photocatalytic reaction active point, thereby improving the separation efficiency of photo-generated charge carriers and photocatalytic performance of the material. The photocatalytic performance of TAP-CN/ZnO series composite materials was generally superior to that of ZnO NWs. When the doping ratio of TAP-CN-30 was 9 wt%, the composite material exhibited the best performance in degrading RhB, with a degradation rate of up to 99.36% within 80 min. The as-prepared TAP-CN/ZnO material exhibited high specific surface area, enhanced visible light absorption by constructing heterojunctions, inhibited photo-generated carrier recombination, and achieved high-performance catalysis. In order to further investigate the photocatalytic degradation activity of RhB, first-order kinetic fitting of the TAP-CN-30/ZnO degradation curve was performed, as shown in Figure 5d. Figure 5d shows the linear fitting curve of −ln(C_t_/C_0_) = f(t), and the corresponding kinetic rate constants are shown in Table 1. C_t_ and C_0_ were the concentrations of RhB in the solution after dark adsorption equilibrium and the corresponding RhB concentrations at time t. According to data analysis, the photocatalytic degradation of RhB followed a first-order kinetic model, and the linear correlation value (R^2^ > 0.90) showed a high fit. The catalytic reaction rate of the composite catalyst was higher than that of ZnO NWs, with 9 wt% TAP-CN/ZnO having the highest K value and the best degradation effect.

The stability of the 9 wt% TAP-CN-30/ZnO catalyst was further investigated through cyclic experiments. The main research focused on the stability of the 9 wt% TAP-CN-30/ZnO cycle, and it can be seen that after three cycles, the degradation rate of RhB can still reach 90.66%. Through cyclic experiments, it can be seen that the material exhibits excellent stability. The photocatalytic mechanism was explored through trapping experiments.

To detect the effects of these active species, we used disodium ethylenediaminetetraacetate (EDTA-2Na), isopropanol (IPA), and benzoquinone (BQ) as capture agents for ·OH, h^+^, and ·O_2_^−^, respectively. By adding sacrificial agents to the photocatalytic system to explore the role of active species in the photodegradation process, the mechanism of photocatalytic reactions could be revealed. The results of photo-generated carrier capture testing in photodegradation elucidate the possible photodegradation mechanism of 9 wt% TAP-CN-30/ZnO. It can be seen that adding BQ as a capture agent for ·O_2_^−^ has a significant inhibitory effect on the degradation of RhB, with a degradation rate reduced from 90.91% to 57.57% in Figure 5f. This indicated that ·O_2_^−^ was the main active species for photocatalytic degradation of RhB; The addition of IPA as a capturing agent for ·OH and EDTA-2Na as a capturing agent for h^+^ had little effect on the degradation of RhB, but had a certain degree of inhibitory effect. The degradation rates decreased from 90.91% to 77.83% and from 90.91% to 79.31%, respectively. There were three active species for the catalytic degradation of Rhb by 9 wt% TAP-CN-30/ZnO: ·OH, h^+^, and ·O_2_^−^, with ·O_2_^−^ being the main active species under visible light.

**Figure 6:**
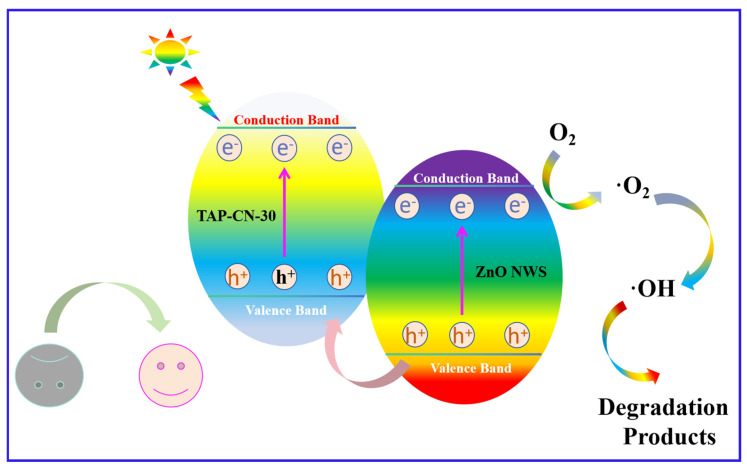
Schematic diagram for the proposed photocatalytic reaction mechanism of 9 wt% TAP-CN-30/ZnO.

The degradation mechanism was shown in the Figure 5f and Figure 6, which was induced by TAP-CN-30 after absorbing visible light π-π* Transition, which transferred excited electrons from HOMO to the lowest unoccupied molecular orbital (LUMO). The LUMO potential of TAP-CN-30 was more negative than the conduction band (CB) edge of ZnO NWs, so the excited electrons on TAP-CN-30 can be directly injected into the CB of ZnO NWs. The VB edge potential of ZnO NWs was more positive than TAP-CN-30, and the holes on the VB of ZnO NWs tended to migrate to TAP-CN-30. It can effectively suppress the photo-induced electron hole pair recombination of ZnO NWs and TAP-CN-30. O_2_ molecules capture photoelectrons on the surface of 9 wt% TAP-CN-30/ZnO and generate superoxide anion radicals (·O_2_^−^). Due to their high activity and inability to penetrate most samples, they mainly desorbed RhB molecules attached to the catalyst surface. Meanwhile, h^+^ reacted with water molecules to produce hydroxyl radicals (·OH). The generated ·OH and h^+^ oxidize organic pollutants into small molecules, such as CO_2_ and H_2_O, in the photocatalytic reaction process described as:

TAP-CN-30 + hv → h^+^ + e^−^ (1)

ZnO NWs + hv → h^+^ + e^−^ (2)

TAP-CN-30 (e^−^_CB_) → ZnO NWs (e^−^_CB_) (3)

ZnO NWs (h^+^_VB_) → TAP-CN-30 (h^+^_VB_) (4)

ZnO NWs (e^−^) + O_2_ → ·O_2_^−^ (5)

·O_2_^−^ + 2H^+^ + e^−^ → H_2_O_2_ (6)

H_2_O_2_ + e^−^ → ·OH + OH^−^ (7)

h^+^ + ·OH + ·O_2_^−^ + pollutants → CO_2_ + H_2_O + degradation intermediate (8)

**Figure 7:**
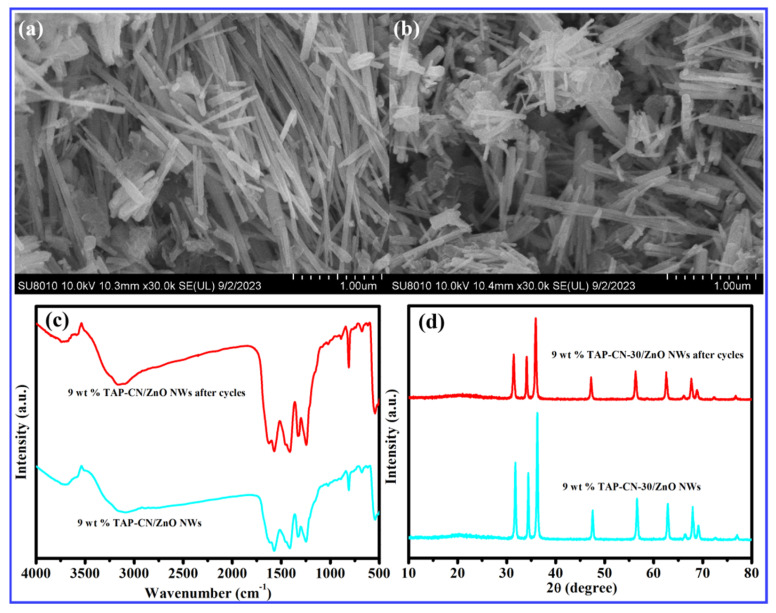
(**a**) The SEM image of 9 wt% TAP-CN-30/ZnO; (**b**) The SEM image of 9 wt% TAP-CN-30/ZnO after cycles; (**c**) XRD patterns of 9 wt% TAP-CN-30/ZnO and 9 wt% TAP-CN-30/ZnO after cycles; (**d**) infrared characterization of 9 wt% TAP-CN-30/ZnO and 9 wt% TAP-CN-30/ZnO after cycles.

Figure 7a,b showed SEM images of the samples before and after cyclic experiments. It could be observed that there was a slight agglomeration phenomenon after 9 wt% TAP-CN-30/ZnO was recycled. It might be the main reason for the slight decrease in catalytic performance. However, the morphology of the catalyst did not change significantly, especially the linear structure of ZnO still uniformly distributed on the surface of g-C_3_N_4_ without detachment. The samples before and after the cycles were tested via FTIR, as shown in Figure 7c. The results showed that there was no change in the position of the infrared absorption peak before and after cycles, and there was no change in the chemical bonds of the sample after cycling degradation. The process of degradation was photocatalytic degradation, and the material structure remained unchanged. To characterize the material structure and crystal structure, the characterization of XRD was performed on the samples before and after cycles in Figure 7d. The results showed that the structure and crystal structure of the prepared sample material did not change. It was further indicating that photocatalytic degradation occurred and the material structure was stable.

## 4. Conclusions

In the paper, Urea and TAP were used to prepare TAP-CN through thermal polymerization, and composite it with ZnO NWs prepared by thermal decomposition method to obtain TAP-CN/ZnO composite materials, forming a heterojunction structure. The microstructure of the composite material was studied through XRD, SEM, TEM, mapping, etc. The photocatalytic performance of TAP-CN/ZnO was studied by the degradation of RhB. The results showed that when TAP doping was 30 mg and the TAP-CN-30 doping ratio was 9 wt%, where 9 wt% TAP-CN-30/ZnO exhibited the best catalytic performance. The degradation rate of RhB solution (20 mg/L) reached 99.36% in 80 min. To study the cyclic stability of 9 wt% TAP-CN-30/ZnO, the results showed good cyclic stability for dye degradation. The photocatalytic mechanism of 9 wt% TAP-CN-30/ZnO through capture experiments was studied. This article provided an idea for constructing conjugated g-C_3_N_4_ systems for applications in the fields of photocatalysis and optoelectronics.

## Figures and Tables

**Table 1:** Kinetic rate constants of the degradation rate of RhB by different catalysts.

	y = ln (C_0_/C_t_)	R^2^	Degradation Rate (%)
ZnO	y = 0.01303x + 0.12447	0.96929	69.18%
3 wt% TAP-CN/ZnO	y = 0.03701x + 0.19386	0.96102	96.23%
6 wt% TAP-CN/ZnO	y = 0.05552x + 0.010009	0.98309	99.20%
9 wt% TAP-CN/ZnO	y = 0.06134x + 0.09746	0.97750	99.36%
12 wt% TAP-CN/ZnO	y = 0.03669x + 0.18199	0.98738	96.67%

## Data Availability

The data presented in this study are available in article.

## References

[1] Li Z., Li R., Jing H., Xiao J., Xie H., Hong F., Ta N., Zhang X., Zhu J., Li C. (2023). Blocking the reverse reactions of overall water splitting on a Rh/GaN-ZnO photocatalyst modified with Al_2_O_3_. Nat. Catal..

[2] Yang R., Fan Y., Zhang Y., Mei L., Zhu R., Qin J., Hu J., Chen Z., Ng Y.H., Voiry D. (2023). 2D transition metal dichalcogenides for photocatalysis. Angew. Chem. Int. Ed..

[3] Zhang Y., Qi M.Y., Tang Z.R., Xu Y.J. (2023). Photoredox-catalyzed plastic waste conversion: Nonselective degradation versus selective synthesis. ACS Catal..

[4] Qiao S., Di M., Jiang J.X., Han B.H. (2022). Conjugated Porous Polymers for Photocatalysis: The Road From Catalytic Mechanism, Molecular Structure to Advanced Applications. EnergyChem.

[5] Zare A., Saadati A., Sheibani S. (2023). Modification of a Z-scheme ZnO-CuO nanocomposite by Ag loading as a highly efficient visible light photocatalyst. Mater. Res. Bull..

[6] Guaraldo T.T., Vakili R., Wenk J., Mattia D. (2023). Highly efficient ZnO photocatalytic foam reactors for micropollutant degradation. Chem. Eng. J..

[7] Wen Y., Chen J., Gao X., Liu W., Che H., Liu B., Ao Y. (2023). Two birds with one stone: Cobalt-doping induces to enhanced piezoelectric property and persulfate activation ability of ZnO nanorods for efficient water purification. Nano Energy.

[8] Wu J., Ke K., Qin N., Lin E., Kang Z., Bao D. (2023). Magnetically retrievable Fe_3_O_4_@ SiO_2_@ ZnO piezo-photocatalyst: Synthesis and multiple catalytic properties. J. Colloid Interface Sci..

[9] Dhiman P., Rana G., Kumar A., Sharma G., Vo D.-V.N., Naushad M. (2022). ZnO-based heterostructures as photocatalysts for hydrogen generation and depollution: A review. Environ. Chem. Lett..

[10] Nguyen N.T.T., Nguyen L.M., Nguyen T.T.T., Liew R.K., Nguyen D.T.C., Tran T.V. (2022). Recent advances on botanical biosynthesis of nanoparticles for catalytic, water treatment and agricultural applications: A review. Sci. Total Environ..

[11] Rai R.S., Bajpai V., Khan M.I., Elboughdiri N., Shanableh A., Luque R. (2023). An eco-friendly approach on green synthesis, bio-engineering applications, and future outlook of ZnO nanomaterial: A critical review. Environ. Res..

[12] Wu Y., Altuner E.E., El Houda Tiri R.N., Bekmezci M., Gulbagca F., Aygun A., Xia C., Van Le Q., Sen F., Karimi-Maleh H. (2023). Hydrogen generation from methanolysis of sodium borohydride using waste coffee oil modified zinc oxide nanoparticles and their photocatalytic activities. Int. J. Hydrogen Energy.

[13] Haounati R., Ighnih H., Malekshah R.E., Alahiane S., Alakhras F., Alabbad E., Alghamdi H., Ouachtak H., Addi A.A., Jada A. (2023). Exploring ZnO/montmorillonite photocatalysts for the removal of hazardous RhB Dye: A combined study using molecular dynamics simulations and experiments. Mater. Today Commun..

[14] Zheng Z., Liang W., Lin R., Hu Z., Wang Y., Lu H., Zhong W., Shen S., Pan Y. (2023). Facile synthesis of zinc indium oxide nanofibers distributed with low content of silver for superior antibacterial activity. Small Struct..

[15] Fatima H., Azhar M.R., Khiadani M., Zhong Y., Wang W., Su C., Shao Z. (2022). Prussian blue-conjugated ZnO nanoparticles for near-infrared light-responsive photocatalysis. Mater. Today Energy.

[16] Wang J., Wang S. (2022). A critical review on *graphitic* carbon nitride (g-C_3_N_4_)-based materials: Preparation, modification and environmental application. Coord. Chem. Rev..

[17] Yang M., Wang P., Li Y., Tang S., Lin X., Zhang H., Zhu Z., Chen F. (2022). Graphene aerogel-based NiAl-LDH/g-C_3_N_4_ with ultratight sheet-sheet heterojunction for excellent visible-light photocatalytic activity of CO_2_ reduction. Appl. Catal. B Environ..

[18] Jiang J., Xiong Z., Wang H., Liao G., Bai S., Zou J., Wu P., Zhang P., Li X. (2022). Sulfur-doped g-C_3_N_4_/g-C_3_N_4_ isotype step-scheme heterojunction for photocatalytic H2 evolution. J. Mater. Sci. Technol..

[19] Liu X., Du Y., Zhao Y., Song X., Jing X., Yu L., Sun M. (2022). 2D Benzodithiophene based conjugated polymer/g-C_3_N_4_ heterostructures with enhanced photocatalytic activity: Synergistic effect of antibacterial carbazole side chain and main chain copolymerization. Appl. Catal. B Environ..

[20] Yuan T., Sun L., Wu Z., Wang R., Cai X., Lin W., Zheng M., Wang X. (2022). Mild and metal-free Birch-type hydrogenation of (hetero) arenes with boron carbonitride in water. Nat. Catal..

[21] Zhang C., Ouyang Z., Yang Y., Long X., Qin L., Wang W., Zhou Y., Qin D., Qin F., Lai C. (2022). Molecular engineering of donor-acceptor structured g-C_3_N_4_ for superior photocatalytic oxytetracycline degradation. Chem. Eng. J..

[22] Ghosh I., Khamrai J., Savateev A., Shlapakov N., Antonietti M., König B. (2019). Organic semiconductor photocatalyst can bifunctionalize arenes and heteroarenes. Science.

[23] Ling G.Z.S., Ng S.F., Ong W.J. (2022). Tailor-engineered 2D cocatalysts: Harnessing electron-hole redox center of 2D g-C_3_N_4_ photocatalysts toward solar-to-chemical conversion and environmental purification. Adv. Funct. Mater..

[24] He W., Liu L., Ma T., Han H., Zhu J., Liu Y., Fang Z., Yang Z., Guo K. (2022). Controllable morphology CoFe_2_O_4_/g-C_3_N_4_ pn heterojunction photocatalysts with built-in electric field enhance photocatalytic performance. Appl. Catal. B Environ..

[25] Li Y., Xia Z., Yang Q., Wang L., Xing Y. (2022). Review on g-C_3_N_4_-based S-scheme heterojunction photocatalysts. J. Mater. Sci. Technol..

[26] Sun H., Wang L., Guo F., Shi Y., Li L., Xu Z., Yan X., Shi W. (2022). Fe-doped g-C_3_N_4_ derived from biowaste material with Fe-N bonds for enhanced synergistic effect between photocatalysis and Fenton degradation activity in a broad pH range. J. Alloys Compd..

[27] Zhang M., Tang L., Zhu Y., Zhang Y., Liu J., Wang J., Feng C., Qiao L., Chen Y. (2023). Conjugated polymers S-scheme homojunction with large internal electric field and matching interface for efficient visible light photocatalytic degradation of ciprofloxacin. J. Clean. Prod..

[28] Sun Y., Wang D., Zhu Y. (2022). Deep degradation of pollutants by perylene diimide supramolecular photocatalyst with unique Bi-planar π-π conjugation. Chem. Eng. J..

[29] Wang Z., Zheng X., Chen P., Li D., Zhang Q., Liu H., Zhong J., Lv W., Liu G. (2022). Synchronous construction of a porous intramolecular DA conjugated polymer via electron donors for superior photocatalytic decontamination. J. Hazard. Mater..

[30] Zhang Q., Chen J., Gao X., Che H., Wang P., Liu B., Ao Y. (2022). Enhanced photocatalytic degradation of bisphenol A by a novel donor-acceptor g-C_3_N_4_: π-π interactions boosting the adsorption and electron transfer behaviors. Sep. Purif. Technol..

[31] Swedha M., Okla M.K., Al-amri S.S., Alaraidh I.A., Al-ghamdi A.A., Mohebaldin A., Abdel-Maksoud M.A., Aufy M., Studenik C.R., Thomas A.M. (2022). Green synthesis of two-electron centre based ZnO/NiCo_2_S_4_ QDs-OVs using Punica granatum fruit peel extract for an exceptional visible light photocatalytic degradation of doxycycline and ciprofloxacin. Chemosphere.

[32] Abubshait H.A., Saad M., Iqbal S., Abubshait S.A., Bahadur A., Raheel M., Alshammari F.H., Alwadai N., Alrbyawi H., Abourehab M.A.S. (2023). Co-doped zinc oxide nanoparticles embedded in Polyvinylalcohol Hydrogel as solar light derived photocatalyst disinfection and removal of coloured pollutants. J. Mol. Struct..

[33] Cong C.Q., Dat N.M., Hai N.D., Nam N.T., An H., Dat T.D., Dat N.T., Phong M.T., Hieu N.H. (2023). Green synthesis of carbon-doped zinc oxide using Garcinia mangostana peel extract: Characterization, photocatalytic degradation, and hydrogen peroxide production. J. Clean. Prod..

[34] Vijayan M., Easwaran G., Sivakumar K., Palanisamy G., Bhuvaneswari K. (2022). Energetic two-dimensional g-C_3_N_4_ nanosheets combined with ZnO nanoparticles as effectual catalyst for degradation of MB dye under UV-Visible-light irradiation. J. Mater. Sci. Mater. Electron..

[35] Guo J., Sun Y., Luo Q., Zhang J., Fang L. (2023). Construction of Z-scheme Ti/Ga co-doped ZnO heterostructure photocatalyst with graphitic carbon nitride for efficient visible-light-driven dye degradation. Environ. Sci. Pollut. Res..

